# Oral Health Assessment for Older Residents in Long-Term Care Facilities Using Video Recording by a Mobile Electronic Device

**DOI:** 10.3390/geriatrics9050135

**Published:** 2024-10-17

**Authors:** Kazuki Ako, Hiroyuki Suzuki, Masataka Watanabe, Hosei Suzuki, Kae Namikawa, Mana Hirayama, Kunihito Yamane, Tomoko Mukai, Yukiko Hatanaka, Junichi Furuya

**Affiliations:** 1 Department of Oral Function Management, Graduate School of Dentistry, Showa University, 2-1-1 Kitasenzoku, Ota-ku, Tokyo 145-8515, Japan; gd21-k001@grad.showa-u.ac.jp (K.A.); m.wata@dent.showa-u.ac.jp (M.W.); gd22-h010@dent.showa-u.ac.jp (H.S.); gd23-k016@dent.showa-u.ac.jp (K.N.); gd22-m019@dent.showa-u.ac.jp (M.H.); y.hatanaka@dent.showa-u.ac.jp (Y.H.); furuyajunichi@gmail.com (J.F.); 2 Division of Oral Function Management, Department of Oral Health Management, School of Dentistry, Showa University, 2-1-1 Kitasenzoku, Ota-ku, Tokyo 145-8515, Japan; kyamane@dent.showa-u.ac.jp (K.Y.); t.mukai@dent.showa-u.ac.jp (T.M.)

**Keywords:** long-term care, oral health, Oral Health Assessment Tool, remote, teledentistry

## Abstract

Background/Objectives: Many older adults who require long-term care need oral health management. However, access to dental care is limited, and connecting older patients with dental professionals is a future challenge. Therefore, the development of a remote oral health assessment system is required. This study aimed to investigate the usefulness of video-based oral health assessments in older adults residing in facilities. Methods: This study comprised 60 older adults residing in facilities who consented to dental home visit treatment by the Department of Oral Function Management at Showa University Dental Hospital between July 2021 and December 2022. The Oral Health Assessment Tool (OHAT) was used to evaluate the oral health status at the facilities by one dentist. The concordance of the oral health assessments conducted by this dentist at the facilities (OHAT-B) was compared with those conducted by the same dentist (OHAT-V1) and two other dentists (OHAT-V2 and V3) using approximately 1 min video recordings of the oral cavity taken with a mobile electronic device. Results: On the OHAT total score, the intraclass correlation coefficient (ICC [1.1]) for OHAT-B and V1 was 0.931; the ICC (2.1) was 0.889 when compared with V2 and 0.788 when compared with V3. Moreover, the comparison between V2 and V3 showed high agreement, with an ICC (2.1) of 0.750. Conclusions: This study revealed that the oral health assessment of older adults residing in facilities using video recordings of the oral cavity taken with a mobile electronic device may be possible, suggesting the possibility of remote oral health assessment.

## 1. Introduction

Aging is progressing globally, and the number of older adults requiring long-term care is predicted to increase [1,2]. In Japan, approximately 30% of older adults aged ≥ 75 years are certified as requiring long-term care and receive medical and nursing care services at home and in facilities [3].

Many older adults requiring long-term care have poor oral health [4,5,6] and need dental treatment [7]. As poor oral health status in older adults requiring long-term care is associated with a systemic condition [8,9,10], implementing appropriate oral health management for these individuals is important. However, older adults requiring long-term care often face difficulties visiting dental clinics because of limitations in physical and cognitive functions [11]. Therefore, oral health management for these individuals is often conducted through dental home visit treatment [12]. This has been shown to improve the oral health of older adults requiring long-term care [13], highlighting the significant role of dental home visit treatment in managing their oral health. In contrast, even in Japan, where there are many older adults requiring long-term care, approximately half of dental clinics do not provide dental home visit treatment [14]. Additionally, in order to receive dental home visit treatment, the older adults requiring long-term care must also bear the associated financial burden [15], leading to a lower frequency of dental care utilization than older adults who do not require care [16].

In situations where it is difficult for dental professionals to directly assess oral health, developing a system that allows the remote evaluation of oral health status is necessary. Using video recordings of the oral environment can provide an oral health assessment comparable to direct assessment [17]. However, as the previous study involved patients admitted to an acute care hospital who could follow instructions and had no cognitive impairments, these individuals were better able to express their oral discomfort, making it easier to connect them with appropriate dental interventions. In addition, it is highly likely that capturing and assessing video footage of oral health conditions in such patients was relatively straightforward. On the other hand, the participants of this study are older adults requiring nursing care in facilities who face greater physical and cognitive limitations. Such patients often struggle to express their oral discomfort, and professionals without dental expertise may have difficulty identifying oral problems, making it challenging to connect these patients to the necessary dental interventions. Therefore, while it is thought that remote oral health assessment using intraoral videos are likely to be effective for older adults requiring long-term care, the usefulness of oral health assessments using intraoral videos for older adults requiring facility-based long-term care remains unclarified.

Therefore, this study aimed to evaluate the usefulness of oral health assessments conducted by dentists using short video recordings of the oral environment captured with a mobile electronic device for older adults residing in facilities requiring long-term care.

## 2. Materials and Methods

### 2.1. Participants

The participants in the study were older adults requiring long-term care who resided in two long-term care facilities and complained about oral discomfort, either independently, through family members, or through facility staff. They provided consent to receive dental home visit treatment from the Department of Oral Function Management at Showa University Dental Hospital between July 2021 and December 2022. Study participants were selected using a convenience sampling method. Those who refused intraoral recordings or had difficulty in maintaining an open mouth or stabilizing their trunk, which hindered intraoral video recording, were excluded. A cross-sectional observational study design was employed for this study. All participants were provided the opportunity to opt out of the study. This study was approved by the Ethics Committee of Showa University (2023-179-B). This study was reported according to the Strengthening the Reporting of Observational Studied in Epidemiology (STROBE) statement (Table S1).

### 2.2. Measurements

At the facilities, a dentist conducted oral health assessments of the participants and used a mobile electronic device (iPhone 14, Apple, Cupertino, CA, USA) to record intraoral videos while ensuring careful protection of personal information. All intraoral video recordings were performed by that dentist. During the intraoral video recording, the participants were seated, and, if necessary, another dentist who was not responsible for the video recording provided assistance, such as retracting the patient’s lips. The intraoral videos were captured using the pre-installed camera application on the device, with settings adjusted to 2× magnification, 720p HD, 30 frames per second, and continuous auto-focus. The flashlight was turned on at all times during the recording. The procedure for intraoral video recording included capturing the lips, frontal view of the anterior teeth, occlusal view of the upper jaw, occlusal view of the lower jaw, left and right buccal mucosa, and tongue. During tongue recording, the participants were instructed to stick out their tongue and move it to the left and right corners of their mouth to ensure that the entire tongue was visible. For participants with dentures, intraoral recordings were made without dentures, followed by extraoral recordings. The participants were asked about the frequency of denture use and the presence of dental pain. For participants with cognitive impairment and communication difficulties, we assessed dentures and dental pain based on the Oral Health Assessment Tool (OHAT) [18]. Specifically, for denture assessments, we also relied on information, such as the frequency of denture use, gathered from caregivers, nurses, and family members. To evaluate dental pain, we focused on identifying non-verbal signs of pain, such as facial expressions and behaviors, to determine its presence or absence. All intraoral video recordings lasted approximately 1 min.

Oral health assessments of the recorded intraoral videos were conducted by three dentists with >3 years of clinical experience in home dental care for older adults requiring long-term care. One of the three dentists recorded the intraoral videos, whereas the remaining two dentists were unfamiliar with the participants’ oral health. These assessments were conducted at least 2 weeks after the facility assessments as outlined in a previous study [17]. Furthermore, the oral health assessments of the recorded intraoral videos were conducted with the participants’ information blinded and the order of the videos was randomized. The evaluators, who regularly use OHAT [18], received approximately 30 min of training on OHAT before assessing the participants’ oral health status from the videos. Calibration for scoring was conducted thoroughly by the evaluators.

### 2.3. Outcomes

The OHAT [18] was used to evaluate oral health status. It includes eight items: lips, tongue, gums and tissues, saliva, natural teeth, dentures, oral cleanliness, and dental pain. These items are categorized into three levels: healthy (0), oral changes (1), and unhealthy (2). The total score ranges from 0 to 16, with higher scores indicating poorer oral health.

In this study, the oral health assessment conducted at a facility based on the OHAT was referred to as the OHAT-B. Oral health assessment conducted using intraoral video recordings based on the OHAT was referred to as the OHAT-V. Among these, the assessment conducted by the dentist who performed the facility evaluation was designated as OHAT-V1, whereas the assessments conducted by the two dentists who did not perform the facility evaluation were designated as OHAT-V2 and OHAT-V3.

### 2.4. Other Variables

Basic patient information, including age, sex, systemic diseases, level of consciousness, communication ability, activities of daily living (ADLs), and level of care needed, was extracted from medical records and conference information. The systemic disease was scored using the Charlson Comorbidity Index [19]. The level of consciousness was evaluated using the Glasgow Coma Scale [20]. Communication ability was categorized as follows: possible (0), partially possible (1), and impossible (2). ADLs were evaluated in three stages: independent (0), requiring partial assistance (1), and requiring full assistance (2). The level of care needed was assessed based on the classification defined by Japan’s public long-term care insurance system [21], ranging from Care Level 1 (requiring some assistance with daily activities, standing, and walking, and showing cognitive decline) to Care Level 5 (requiring comprehensive care for all daily activities). A higher level of care indicates a greater need for care.

### 2.5. Statistical Analyses

To examine the reliability of oral health assessments performed by the same evaluator at the facility and on video, we compared the OHAT-B and OHAT-V1. For the total score, the intraclass correlation coefficient (ICC1.1) was calculated. For the OHAT sub-items, the weighted kappa coefficient and percentage agreement were computed. To evaluate the reliability between the facility-based oral health assessment and video-based assessments conducted by two dentists who did not perform the facility evaluation, comparisons were made between the OHAT-B and OHAT-V2, and OHAT-V3, respectively. Additionally, to assess the reliability of the video-based oral health evaluations, the OHAT-V2 and OHAT-V3 were compared. For these comparisons, the ICC (ICC2.1) was used for the total score, and the weighted kappa coefficient and percentage agreement were calculated for the OHAT sub-items. Statistical analyses were performed using SPSS version 28 (IBM Japan), with a significance level of 5%.

## 3. Results

### 3.1. Characteristics of the Participants

Sixty institutionalized older adults (mean age, 86.1 ± 7.7 years; 18 men and 42 women) participated in this study. The participants’ characteristics are presented in Table 1.

Dementia and cerebrovascular disease were observed in 70% and 35% of the participants, respectively. Communication ability equivalent to that of healthy individuals was observed in 50% of the participants. Regarding ADLs, >83% of the participants required assistance. The most common level of care needed was Level 5, which was observed in 35% of the participants.

### 3.2. Oral Health Status Assessment at the Facilities and via Intraoral Video Recording

The results of the oral health status assessments conducted at the facilities (OHAT-B) and via intraoral video recordings (OHAT-V1, V2, and V3) are shown in Table 2.

**Figure 1:**
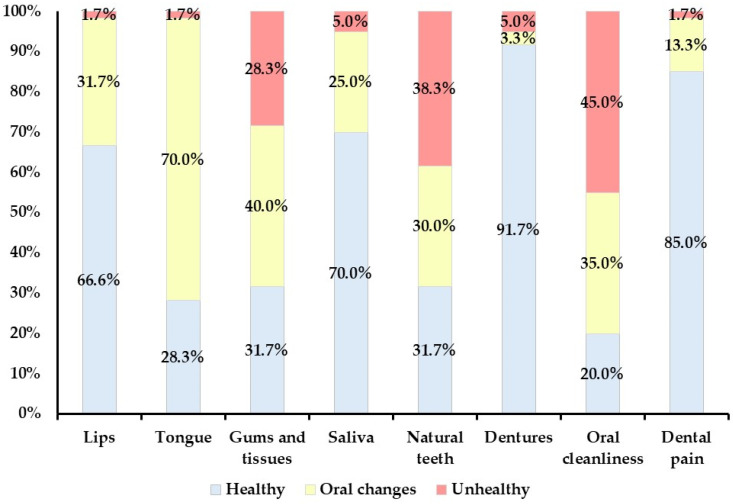
Distribution of sub-items in Oral Health Assessment Tool assessment by dentists at the facility (*N* = 60).

The median total score for the OHAT-B was 5, whereas those for the OHAT-V1, V2, and V3 were 5, 5.5, and 5, respectively. The distribution of the OHAT-B sub-item scores is shown in Figure 1.

Regarding the OHAT sub-items, 66.7% and 70.0% of participants had a healthy status of lips and saliva, respectively. For dentures, 91.7% of participants were assessed as having a healthy status, and most participants were able to use dentures appropriately. On the other hand, 70.0% and 40.0% of participants were assessed as having oral changes related to the tongue and gums and tissues, respectively. Moreover, 38.3% and 45.0% of participants were assessed as unhealthy concerning their natural teeth and oral cleanliness, respectively. The most common reasons for participants being classified as having oral changes or an unhealthy status included tongue coating, gingival swelling, dental caries, residual roots, food residue, and plaque retention.

### 3.3. Reliability of Oral Health Status Assessment Using Intraoral Video Recordings

The ICC, weighted kappa coefficient, and percent agreement between the OHAT-B and OHAT-V1, V2, and V3 are shown in Table 3.

When the OHAT-B and OHAT-V1 were evaluated by the same rater, the ICC of the OHAT total score (1.1) was 0.931 (95% confidence interval [CI], 0.888–0.958), indicating almost perfect agreement. The weighted kappa coefficients for all the sub-items were >0.7, indicating substantial agreement.

When the OHAT-B was compared with OHAT-V2, the ICC (2.1) of the OHAT total score was 0.889 (95% CI, 0.819–0.932), indicating almost perfect agreement. The weighted kappa coefficients for all the sub-items were >0.7, indicating substantial agreement.

When the OHAT-B was compared with OHAT-V3, the ICC (2.1) of the OHAT total score was 0.788 (95% CI, 0.669–0.867), indicating substantial agreement. The weighted kappa coefficient of saliva was moderately consistent, but the other sub-items showed substantial agreement.

The ICC, weighted kappa coefficients, and percent agreement between the OHAT-V2 and OHAT-V3 are shown in Table 4.

The ICC (2.1) of the OHAT total score was 0.750 (95% CI, 0.610–0.844), indicating substantial agreement. The weighted kappa coefficients for saliva were moderately consistent, whereas those for the other categories showed substantial agreement.

## 4. Discussion

The results of this study indicate that, when assessing the oral health status of institutionalized older adults requiring long-term care, there was a high reliability in the OHAT total scores, both within the same evaluator and between different evaluators, whether the assessment was performed directly in person by a dentist based on an oral examination at the facilities or by using short videos recorded on a mobile electronic device. All OHAT sub-items showed moderate agreement. These findings suggest that, even for institutionalized older adults requiring long-term care, it may be possible to remotely evaluate their oral health status using intraoral videos recorded on small portable devices. The provision of dental services to institutionalized older adults requiring long-term care is limited [16], and the potential use of intraoral videos recorded on mobile electronic devices as a tool to connect these individuals with dental professionals is a clinically significant finding.

In this study, the total OHAT scores evaluated from intraoral videos demonstrated almost perfect agreement with the scores assessed at the facilities. The OHAT total score serves as an effective indicator for determining the need for oral health management [22]. This suggests that, even when a dentist cannot directly assess the oral health status of older adults, intraoral videos enable dental professionals to assess the need for intervention either online in real time or on demand at a later time from a different location. Furthermore, many OHAT sub-items showed high agreement between evaluations conducted at the facility and those based on intraoral videos. This finding aligns with previous studies indicating that oral hygiene status and assessments related to restorative prosthetics and dentures can be accurately evaluated remotely, similar to face-to-face assessments [23,24]. However, the evaluation of saliva showed lower agreement compared to other items, likely due to the difficulties in assessing the viscosity and physical properties of saliva from videos. The information obtained from intraoral videos is limited and does not completely match the findings obtained with specialized onsite equipment. Thus, it cannot replace actual intraoral examinations. However, the good agreement between facility-based evaluations and intraoral videos suggests that intraoral videos can provide valuable diagnostic information for dentists.

Since the onset of the COVID-19 pandemic, telemedicine has gained attention in the field of dentistry [25]. These telemedicine services include oral health education [26,27] supported by mobile applications and remote oral assessment [28,29], with research targeting older adults in long-term care. However, reports indicate challenges with mobile applications used for remote oral health education, particularly regarding older adults’ ability to use them effectively [26,27]. In contrast, remote oral assessments may be effective in alleviating access barriers to dental services for older adults requiring long-term care. For example, photos taken with smartphones can be used to evaluate oral and denture hygiene statuses [23]. Moreover, videos taken using endoscopes can accurately diagnose the dental pathology, the chewing ability, and the status of prosthetic treatment [24]. These findings suggest the effectiveness of remote dental diagnosis using imaging devices in institutionalized older adults requiring long-term care. Consistent with these reports, this study demonstrates that high-precision oral health assessments are possible using intraoral videos. The mobile electronic device used in this study is more widely available than endoscopes, making it easily usable in many medical and caregiving settings. When performing intraoral recording, ensuring adequate lighting is essential [30], and attention must be given to areas that may be difficult to focus on, depending on the shooting location. To address these issues, intraoral photography was performed using the flashlight and autofocus functions of a mobile electronic device. The use of such mobile electronic devices equipped with high mobility, flashlights, and autofocus functions may be effective in environments where patients have difficulty following instructions or where there are positional constraints.

In this study, 70% of participants had dementia, and 50% had communication difficulties. Dementia is one of the causes requiring long-term care [2], and many long-term care facility residents with cognitive impairments experience declines in ADLs, voluntary hygienic behaviors, and overall oral hygiene [31]. Additionally, there are often instances where oral assessment and care are refused, complicating oral management for care staff [32,33]. For these institutionalized older adults, although active management by dental professionals is essential for maintaining good oral health, access to dental care is often limited. The results of this study demonstrate that intraoral videos can accurately evaluate the oral health status of patients with dementia or communication difficulties. This suggests that remote oral health status evaluation using intraoral videos could help improve access to dental care for older adults in need of long-term care.

This study has some limitations. First, the person capturing the videos and the assistant holding the lips back were dentists. Some participants in this study were resistant or had difficulty following instructions, which led to challenges in directing them during the intraoral video recordings. Dentists are naturally accustomed to conducting oral examinations and are likely to understand what types of videos are needed to evaluate oral health status effectively. Therefore, it is possible that the intraoral videos recorded in this study are particularly suitable for facilitating oral health assessments. Given that, in environments where a dentist is not present, caregiving staff often play a primary role in managing the oral health of facility residents, investigating whether oral health assessments can be conducted using videos taken by the facility staff or other professionals is necessary. Second, this study was restricted to institutionalized older adults requiring care. Although older adults receiving domiciliary care tend to experience deteriorating oral health similar to those in institutionalized ones [4], they have fewer opportunities to receive dental treatment compared with their institutionalized counterparts [34]. Therefore, future studies should also target older adults receiving domiciliary care. However, as the patient characteristics of those older adults may not significantly differ from those of institutionalized older adults requiring care, the findings of this study can potentially be applicable to the latter group. Finally, in this study, those who refused intraoral recordings or had difficulty in maintaining an open mouth or stabilizing their trunk, which prevented the recording of intraoral videos, were excluded. This selection bias is a noted limitation of this study. It is likely that these excluded patients had poorer oral health, as conducting an oral examination on them would be challenging. Including these patients in this could have clarified the oral health characteristics of patients unable to undergo assessments using intraoral videos. However, as the purpose of this study was to evaluate the usefulness of oral health assessment using intraoral videos for older adults requiring long-term care, the oral health of participants who met the eligibility criteria was assessed in this study.

## 5. Conclusions

When dentists use short intraoral videos recorded on a mobile electronic device to assess the oral health status of institutionalized older adults requiring care, there is a high level of agreement with oral health assessments based on in-person oral examinations conducted at the facility. These findings may suggest the usefulness of the remote oral health status evaluation of institutionalized older adults requiring long-term care using intraoral videos recorded on small portable devices.

## Figures and Tables

**Table 1:** Participant characteristics.

		Mean ± SD	Median	n	%
Age		86.1 ± 7.65	88	60	
Sex	Male			18	30.0
	Female			42	70.0
Systemic disorder	Dementia			42	70.0
	High blood pressure			21	35.0
	Cerebrovascular disease			21	35.0
	Bone fracture			11	18.3
	Disuse syndrome			10	16.7
	Heart disease			9	15.0
	Diabetes mellitus			9	15.0
	Parkinson’s disease			7	11.7
	Aspiration pneumonia			5	8.3
	Others			5	8.3
CCI		2.3 ± 1.25	2.0		
GCS		12.9 ± 2.61	14.0		
Communication ability	0			30	50.0
	1			19	31.7
	2			11	18.3
ADLs	0			10	16.7
	1			26	43.3
	2			24	40.0
Level of care needed	1			3	5.0
	2			6	10.0
	3			15	25.0
	4			15	25.0
	5			21	35.0

Abbreviations: ADLs, activities of daily living; CCI, Charlson Comorbidity Index; GCS, Glasgow Coma Scale; SD, standard deviation.

**Table 2:** Results of OHAT total score and sub-item scores for the OHAT-B, an oral health assessment conducted at the institutions, and the OHAT-V1, V2, and V3, oral health assessments made using intraoral video recordings.

Category	B	V1	V2	V3
	Mean ± SD	Mean ± SD	Mean ± SD	Mean ± SD
	Median	Median	Median	Median
Lips	0.4 ± 0.5	0.4 ± 0.5	0.4 ± 0.6	0.3 ± 0.5
	0.0	0.0	0.0	0.0
Tongue	0.7 ± 0.5	0.7 ± 0.5	0.7 ± 0.4	0.7 ± 0.5
	1.0	1.0	1.0	1.0
Gums and tissues	1.0 ± 0.8	0.8 ± 0.8	1.0 ± 0.7	0.8 ± 0.8
	1.0	1.0	1.0	1.0
Saliva	0.4 ± 0.6	0.4 ± 0.6	0.5 ± 0.5	0.3 ± 0.5
	0.0	0.0	0.0	0.0
Natural teeth	1.1 ± 0.8	1.0 ± 0.8	1.2 ± 0.8	1.0 ± 0.8
	1.0	1.0	1.0	1.0
Dentures	0.1 ± 0.5	0.1 ± 0.4	0.1 ± 0.5	0.2 ± 1.1
	0.0	0.0	0.0	0.0
Oral cleanliness	1.3 ± 0.8	1.3 ± 0.8	1.2 ± 0.7	1.2 ± 0.8
	1.0	2.0	1.0	1.5
Dental pain	0.2 ± 0.4	0.2 ± 0.4	0.1 ± 0.3	0.2 ± 0.4
	0.0	0.0	0.0	0.0
Total score	5.0 ± 2.3	5.0 ± 2.5	5.3 ± 2.1	4.8 ± 2.3
	5.0	5.0	5.5	5.0

Abbreviations: SD, standard deviation; OHAT, Oral Health Assessment Tool.

**Table 3:** Intraclass correlation coefficients, weighted kappa coefficients, and percent agreement between OHAT-B, the oral health status assessment conducted at the institutions, and OHAT-V1, V2, and V3, the oral health status assessments made using the intraoral video recordings.

Category	B-V1		B-V2		B-V3	
	Percent Agreement	Weighted Kappa Coefficients (95% CI)	Percent Agreement	Weighted Kappa Coefficients (95% CI)	Percent Agreement	Weighted Kappa Coefficients (95% CI)
Lips	88	0.787 * (0.626–0.947)	87	0.768 * (0.612–0.924)	95	0.903 * (0.792–1.015)
Tongue	90	0.782 * (0.610–0.954)	87	0.686 * (0.492–0.880)	98	0.964 * (0.894–1.034)
Gums and tissues	78	0.793 * (0.665–0.921)	75	0.769 * (0.655–0.882)	73	0.751 * (0.617–0.885)
Saliva	85	0.773 * (0.624–0.922)	77	0.629 * (0.449–0.808)	77	0.572 * (0.396–0.748)
Natural teeth	85	0.891 * (0.821–0.961)	75	0.816 * (0.726–0.906)	80	0.857 * (0.777–0.936)
Dentures	95	0.754 * (0.429–1.079)	95	0.881 * (0.714–1.047)	95	0.918 * (0.785–1.051)
Oral cleanliness	85	0.873 * (0.787–0.959)	85	0.864 * (0.776–0.952)	75	0.808 * (0.708–0.907)
Dental pain	97	0.903 * (0.760–1.046)	93	0.769 * (0.583–0.956)	95	0.850 * (0.662–1.038)
	**Intraclass correlation coefficients (1.1)** **(95% CI)**		**Intraclass correlation coefficients (2.1)** **(95% CI)**		**Intraclass correlation coefficients (2.1)** **(95% CI)**	
Total score	0.931 ** (0.888–0.958)		0.889 ** (0.819–0.932)		0.788 ** (0.669–0.867)	

* *p* < 0.05, weighted kappa coefficients. ** *p* < 0.05, intraclass correlation coefficients. Abbreviations: CI, confidence interval; OHAT, Oral Health Assessment Tool.

**Table 4:** Intraclass correlation coefficient, weighted kappa coefficient, and % agreement between OHAT-V2 and OHAT-V3, the OHAT assessments made using the intraoral video taken by dentists who did not know the oral environment.

Category	OHAT V2–V3	
	Percent Agreement	Weighted Kappa Coefficients (95% CI)
Lips	85	0.738 * (0.571–0.905)
Tongue	88	0.730 * (0.550–0.910)
Gums and tissues	63	0.681 * (0.554–0.808)
Saliva	73	0.494 * (0.296–0.692)
Natural teeth	72	0.720 * (0.572–0.868)
Dentures	98	0.687 * (0.228–1.145)
Oral cleanliness	70	0.755 * (0.642–0.868)
Dental pain	92	0.699 * (0.480–0.918)
	**Intraclass correlation coefficient (2.1) (95% CI)**	
Total score	0.750 ** (0.610–0.844)	

* *p* < 0.05, weighted kappa coefficients; ** *p* < 0.05, intraclass correlation coefficients. Abbreviations: CI, confidence interval; OHAT, Oral Health Assessment Tool.

## Data Availability

The data supporting the findings of this study are available from the corresponding author upon reasonable request.

## Supplementary Materials

The following supporting information can be downloaded at: https://www.mdpi.com/article/10.3390/geriatrics9050135/s1, Table S1: Strengthening the Reporting of Observational Studied in Epidemiology (STROBE) statement.

## References

[1] Xue Q.L., Bandeen-Roche K., Varadhan R., Zhou J., Fried L.P. (2008). Initial manifestations of frailty criteria and the development of frailty phenotype in the Women’s Health and Aging Study II. J. Gerontol. A Biol. Sci. Med. Sci..

[2] Koller D., Schön G., Schäfer I., Glaeske G., van den Bussche H., Hansen H. (2014). Multimorbidity and long-term care dependency—A five-year follow-up. BMC Geriatr..

[3] Nishino T. (2017). Quantitative properties of the macro supply and demand structure for care facilities for elderly in Japan. Int. J. Environ. Res. Public Health.

[4] Tanaka K., Tominaga T., Kikutani T., Sakuda T., Tomida H., Tanaka Y., Mizukoshi A., Ichikawa Y., Ozeki M., Takahashi N. (2024). Oral status of older adults receiving home medical care: A cross-sectional study. Geriatr. Gerontol. Int..

[5] Park Y.-H., Han H.-R., Oh B.-M., Lee J., Park J.-A., Yu S.J., Chang H. (2013). Prevalence and associated factors of dysphagia in nursing home residents. Geriatr. Nurs..

[6] Yoon M.N., Ickert C., Slaughter S.E., Lengyel C., Carrier N., Keller H. (2018). Oral Health status of long-term care residents in Canada: Results of a national cross-sectional study. Gerodontology.

[7] Gerritsen P., Cune M., van der Bilt A., Abbink J., de Putter C. (2015). Effects of integrated dental care on oral treatment needs in residents of nursing homes older than 70 years. Spec. Care Dent..

[8] Liu S., Guo Y., Hu Z., Zhou F., Li S., Xu H. (2023). Association of oral status with frailty among older adults in nursing homes: A cross-sectional study. BMC Oral Health.

[9] Fukuyama Y., Komiyama T., Ohi T., Hattori Y. (2024). Association between oral health and nutritional status among older patients requiring long-term care who received home-visit dental care. J. Oral Sci..

[10] Dewake N., Hashimoto H., Nonoyama T., Nonoyama K., Shimazaki Y. (2020). Posterior occluding pairs of teeth or dentures and 1-year mortality in nursing home residents in Japan. J. Oral Rehabil..

[11] Beaven A., Marshman Z. (2024). Barriers and facilitators to accessing oral healthcare for older people in the UK: A scoping review. Br. Dent. J..

[12] Komulainen K., Ylöstalo P., Syrjälä A., Ruoppi P., Knuuttila M., Sulkava R., Hartikainen S. (2012). Preference for dentist’s home visits among older people. Community Dent. Oral Epidemiol..

[13] Tanaka K., Kikutani T., Takahashi N., Tohara T., Furuya H., Ichikawa Y., Komagata Y., Mizukoshi A., Ozeki M., Tamura F. (2024). A prospective cohort study on factors related to dental care and continuation of care for older adults receiving home medical care. Odontology.

[14] Nomura Y., Okada A., Kakuta E., Otsuka R., Saito H., Maekawa H., Daikoku H., Hanada N., Sato T. (2020). Workforce and contents of home dental care in Japanese insurance system. Int. J. Dent..

[15] Ni S.C., Thomas C., Yonezawa Y., Hojo Y., Nakamura T., Kobayashi K., Sato H., Da Silva J.D., Kobayashi T., Ishikawa-Nagai S. (2022). Comprehensive assessment of the universal healthcare system in dentistry Japan: A retrospective observational study. Healthcare.

[16] Czwikla J., Rothgang H., Schwendicke F., Hoffmann F. (2023). Dental care utilization among home care recipients, nursing home residents, and older adults not in need of long-term care: An observational study based on German insurance claims data. J. Dent..

[17] Yanagihara Y., Suzuki H., Furuya J., Nakagawa K., Yoshimi K., Seto S., Shimizu K., Tohara H., Minakuchi S. (2024). Usefulness of oral health assessment performed by multiple professionals using a short video recording acquired with a tablet device. J. Dent. Sci..

[18] Chalmers J.M., King P.L., Spencer A.J., Wright F.A., Carter K.D. (2005). The oral health assessment tool—Validity and reliability. Aust. Dent. J..

[19] Charlson M.E., Pompei P., Ales K.L., MacKenzie C.R. (1987). A new method of classifying prognostic comorbidity in longitudinal studies: Development and validation. J. Chronic Dis..

[20] Jennett B., Teasdale G. (1977). Aspects of coma after severe head injury. Lancet.

[21] Konishi T., Inokuchi H., Yasunaga H. (2023). Services in public long-term care insurance in Japan. Ann. Clin. Epidemiol..

[22] Suzuki H., Furuya J., Nakagawa K., Hidaka R., Nakane A., Yoshimi K., Shimizu Y., Saito K., Itsui Y., Tohara H. (2023). Factors influencing the selection of oral healthcare providers in multidisciplinary Nutrition Support Team for malnourished inpatients: A cross-sectional study. J. Oral Rehabil..

[23] Bleiel D., Rott T., Scharfenberg I., Wicht M.J., Barbe A.G. (2023). Use of smartphone photos to document the oral care status of nursing home residents. Gerodontology.

[24] Queyroux A., Saricassapian B., Herzog D., Müller K., Herafa I., Ducoux D., Marin B., Dantoine T., Preux P.-M., Tchalla A. (2017). Accuracy of teledentistry for diagnosing dental pathology using direct examination as a gold standard: Results of the Tel-e-dent study of older adults living in nursing homes. J. Am. Med. Dir. Assoc..

[25] Liu T.C., Chang Y.C. (2024). A bibliometric analysis of teledentistry published in the category of dentistry, oral surgery and medicine. J. Dent. Sci..

[26] Wang Q., Liu J., Zhou L., Tian J., Chen X., Zhang W., Wang H., Zhou W., Gao Y. (2022). Usability evaluation of mHealth apps for elderly individuals: A scoping review. BMC Med. Inform. Decis. Mak..

[27] Chau R.C.W., Thu K.M., Chaurasia A., Hsung R.T.C., Lam W.Y. (2023). A systematic review of the use of mHealth in oral health education among older adults. Dent. J..

[28] Fonseca B.B., Perdoncini N.N., da Silva V.C., Gueiros L.A.M., Carrard V.C., Lemos C.A., Schussel J.L., Amenábar J.M., Torres-Pereira C.C. (2022). Telediagnosis of oral lesions using smartphone photography. Oral Dis..

[29] Pandey P., Jasrasaria N., Bains R., Singh A., Manar M., Kumar A. (2023). The efficacy of dental caries telediagnosis using smartphone: A diagnostic study in geriatric patients. Cureus.

[30] Ahmad I. (2009). Digital dental photography. part 5: Lighting. Br. Dent. J..

[31] Rapp L., Sourdet S., Vellas B., Lacoste-Ferré M.H. (2017). Oral Health and the frail elderly. J. Frailty Aging.

[32] Chalmers J.M. (2000). Behavior management and communication strategies for dental professionals when caring for patients with dementia. Spec. Care Dent..

[33] Chalmers J., Pearson A. (2005). Oral hygiene care for residents with dementia: A literature review. J. Adv. Nurs..

[34] Ishimaru M., Ono S., Morita K., Matsui H., Yasunaga H. (2019). Domiciliary dental care among homebound older adults: A nested case-control study in Japan. Geriatr. Gerontol. Int..

